# The Efficacy and Safety of Myelophil, an Ethanol Extract Mixture of Astragali Radix and Salviae Radix, for Chronic Fatigue Syndrome: A Randomized Clinical Trial

**DOI:** 10.3389/fphar.2019.00991

**Published:** 2019-09-10

**Authors:** Jin-Yong Joung, Jin-Seok Lee, Jung-Hyo Cho, Dong-Soo Lee, Yo-Chan Ahn, Chang-Gue Son

**Affiliations:** ^1^Liver and Immunology Research Center, Oriental Medical Collage of Daejeon University, Daejeon, South Korea; ^2^Department of Internal Medicine, Daejeon St. Mary’s Hospital of Catholic University, Daejeon, South Korea; ^3^Department of Health Service Management, Daejeon University, Daejeon, South Korea

**Keywords:** chronic fatigue syndrome, systemic exertion intolerance disease, Myelophil, *Astragali Radix*, *Salviae Mitiorrhizae Radix*, clinical trial

## Abstract

**Background:** There is a strong demand for therapeutics to treat chronic fatigue syndrome (CFS), although there are limitations. Myelophil, which is a combination of extracts from *Astragali Radix* and *Salviae Miltiorrhizae Radix*, has been clinically used to treat fatigue-related disorders in South Korea. We conducted a randomized controlled clinical trial of Myelophil in patients with CFS and evaluated its efficacy and safety in two hospitals.

**Methods:** We enrolled 98 participants (M: 38, F: 60) with CFS in a phase 2 trial of oral Myelophil (2 g daily) or placebo for 12 weeks. The primary end point was a change in the Chalder fatigue scale, as scored by a numeric rating scale (NRS). The secondary end points included changes in the visual analogue scale, fatigue severity scale (FSS), and 36-item short-form health survey (SF-36). Biomarkers of oxidative stress and cytokines were evaluated by blood tests.

**Results:** Ninety-seven participants (48 in the Myelophil group and 49 in the placebo group) completed the trial. An analysis of all participants showed that Myelophil slightly improved fatigue symptoms compared with those of the placebo, but this effect was not statistically significant (p > 0.05 for the NRS, VAS, FSS, and SF-36). By contrast, an analysis of the subpopulation (53 participants, M: 24, F: 29) with severe symptoms (≥63, median NRS value of total participants) showed a statistically significant improvement in fatigue symptoms in the Myelophil group compared with the placebo (p < 0.05 for NRS, FSS, and SF-36). There were no significant changes in the biomarkers for oxidative stress and cytokines before or after the treatment. No Myelophil-related adverse response was observed during the trial.

**Conclusion:** These results support the hypothesis that Myelophil can be a therapeutic candidate to manage CFS and provide the rationale for its progression to a phase 3 clinical trial.

**Clinical Trial Registration:** www.ClinicalTrials.gov, identifier KCT0002317.

## Introduction

Chronic fatigue syndrome (CFS) is a complicated disease that is characterized by extreme fatigue with substantial symptoms that are associated with neurological, cognitive, and immune abnormalities for at least 6 months (Wyller, 2007; Moss-Morris et al., 2013). CFS cannot be explained by any medical examinations, and it impairs the patient quality of life by physical, social, and occupational factors (Jorgensen, 2008). Specifically, to emphasize the medical impact of CFS, the Institute of Medicine in the USA suggested changing the name of CFS to systemic exertion intolerance disease without the word “fatigue” and asked the US government to accelerate the research progress for CFS treatment by raising funds in 2015 (Committee on the Diagnostic Criteria for Myalgic Encephalomyelitis/Chronic Fatigue Syndrome et al., 2015). The prevalence of CFS in developed countries and among the working age population is generally higher than that of developing countries and the aged population (Skapinakis et al., 2003). It is estimated that 0.2–2.6% of the world’s population suffers from CFS (Reyes et al., 2003; Prins et al., 2006).

The underlying mechanism of CFS remains unclear. However, scientific studies suggest that an abnormality of the central nervous system (CNS) is involved in CFS (Chen et al., 2008; Ferrero et al., 2017). Altered serotonergic and muscarinic neurotransmitter systems (Katafuchi et al., 2006; Yamamoto et al., 2012), immunological dysfunction (Kennedy et al., 2005; Morris et al., 2013; Nijs et al., 2014), and disruption of the hypothalamic–pituitary–adrenal (HPA) axis (Papadopoulos and Cleare, 2011; Tomas et al., 2013) were reported to contribute to CNS impairment in CFS patients. Based on this finding, antidepressants, immunomodulatory agents, and corticosteroids were studied as therapeutic agents, but no clear effects of these treatments were observed (Castro‐Marrero et al., 2017). Only cognitive behavioral and exercise therapies have been recommended, although they only have partial benefits (Price et al., 2008; Larun et al., 2016). However, previous recommendations of these therapies that were based on a large-scale clinical study (called the “Pacing, graded activity, and cognitive behaviour therapy; a randomised evaluation” trial) were abandoned or revised in both the USA and UK due to serious criticism by both scientists and patients (Geraghty, 2017; Vink, 2017).

To date, two phase 3 clinical trials have been performed for CFS using the following medications: 1) a monoclonal anti-CD20 antibody, Rituximab, and 2) the nucleic acid compounds, Rintatolimod. Both of these therapies, however, did not provide more credible evidence of efficacy than the placebo (Strayer et al., 2012; Smith et al., 2014; Günther et al., 2019). These disappointing results demand the additional trials for the development of an effective therapy for CFS. Myelophil is a 1:1 mixture of the 30% ethanol extracts of *Astragali radix* [scientific name: *Astragalus mongholicus* var. *dahuricus* (DC.) Podlech] and *Salviae radix* (scientific name: *Salvia miltiorrhiza* Bunge, 1833) and is clinically used to treat patients with chronic fatigue-related disorders (Shin et al., 2008; Lee et al., 2015). According to traditional Chinese medicine, *Astragali radix* and *Salviae radix* are the representative herbs providing support for two essential components of the human body, *Qi* and *blood*, respectively. *Qi* means the circulating life energy behind every activities and processes, including metabolism and growth of the human body, and *blood* is a vital substance responsible for the nourishment of the body. The deficiency of *Qi* and *blood* is known to be associated with physical and mental fatigue (Kim et al., 2016). In preclinical studies, Myelophil showed an anti-fatigue effect and prevented CNS damage through potent anti-inflammatory, antioxidant, and HPA axis modulating effects (Kim et al., 2012; Lee et al., 2012; Kim et al., 2013; Kim et al., 2014; Lee et al., 2014). This effect has also been demonstrated in a clinical study of patients with idiopathic chronic fatigue (ICF) (Cho et al., 2009). Previous animal studies have proven the safety of Myelophil in both repeated toxicity and genotoxicity studies (Jung et al., 2009; Lee et al., 2018).

This phase 2, randomized, placebo-controlled trial was aimed at evaluating the efficacy and safety of Myelophil and providing essential clinical data for the following phase 3 trial.

## Materials and Methods

### Trial Oversight

The trial was supported by the government grants from Ministry of Health & Welfare and Ministry of Education, Science and Technology, Republic of Korea. The protocol was approved by the institutional review board at each center (institutional review board number: DJDSKH-17-DR-03 in Daejeon Korean Medicine Hospital, DIRB-00139-3 in Daejeon St. Mary’s Hospital). An independent medical monitor, MEDICAL excellence, ensured that the trial was conducted according to the protocol and maintained the data. Data were analyzed by a medical statistics specialist. The test drug and matching placebo were provided at no cost by Kyongbang Pharm. Co., Ltd. Kyongbang Pharmacy also provided less than 5% of the total trial funding through an agreement with the Ministry of Health & Welfare. There was no confidentiality agreement between the authors and Kyongbang Pharm. All participants provided voluntary written informed consent. This clinical trial was conducted in accordance with the International Council for Harmonisation. This study is registered at https://cris.nih.go.kr/ with identifier number KCT0002317.

### Patients

The key eligibility criteria were participants between the ages of 18 and 65 and a diagnosis of CFS, according to the definition of the US Centers for Disease Control and Prevention (CDC) (Brurberg et al., 2014), which requires clinically evaluated, unexplained, persistent, or relapsing chronic fatigue. Additionally, study inclusion required the concurrent occurrence of four or more of the following symptoms, all of which must have persisted or recurred during 6 or more consecutive months of illness and must not have predated the fatigue: self-reported impairment in short-term memory or concentration; sore throat; cervical or axillary lymphadenopathy; muscle pain; multi-joint pain without joint swelling or redness; headaches of a new type, pattern, or severity; unrefreshing sleep; and post-exertional malaise lasting more than 24 hours. All other known causes of chronic fatigue must have been ruled out.

The key exclusion criteria were participants who required continuous medication for other illnesses or suffered from diseases that induced chronic fatigue within the past 6 months. Such disease include anemia; liver, kidney, and thyroid dysfunction; depression; and anxiety disorders. Details of inclusion and exclusion criteria are provided in the **Supplementary Tables 1 and 2**.

### Trial Design and Treatments

**Figure 1:**
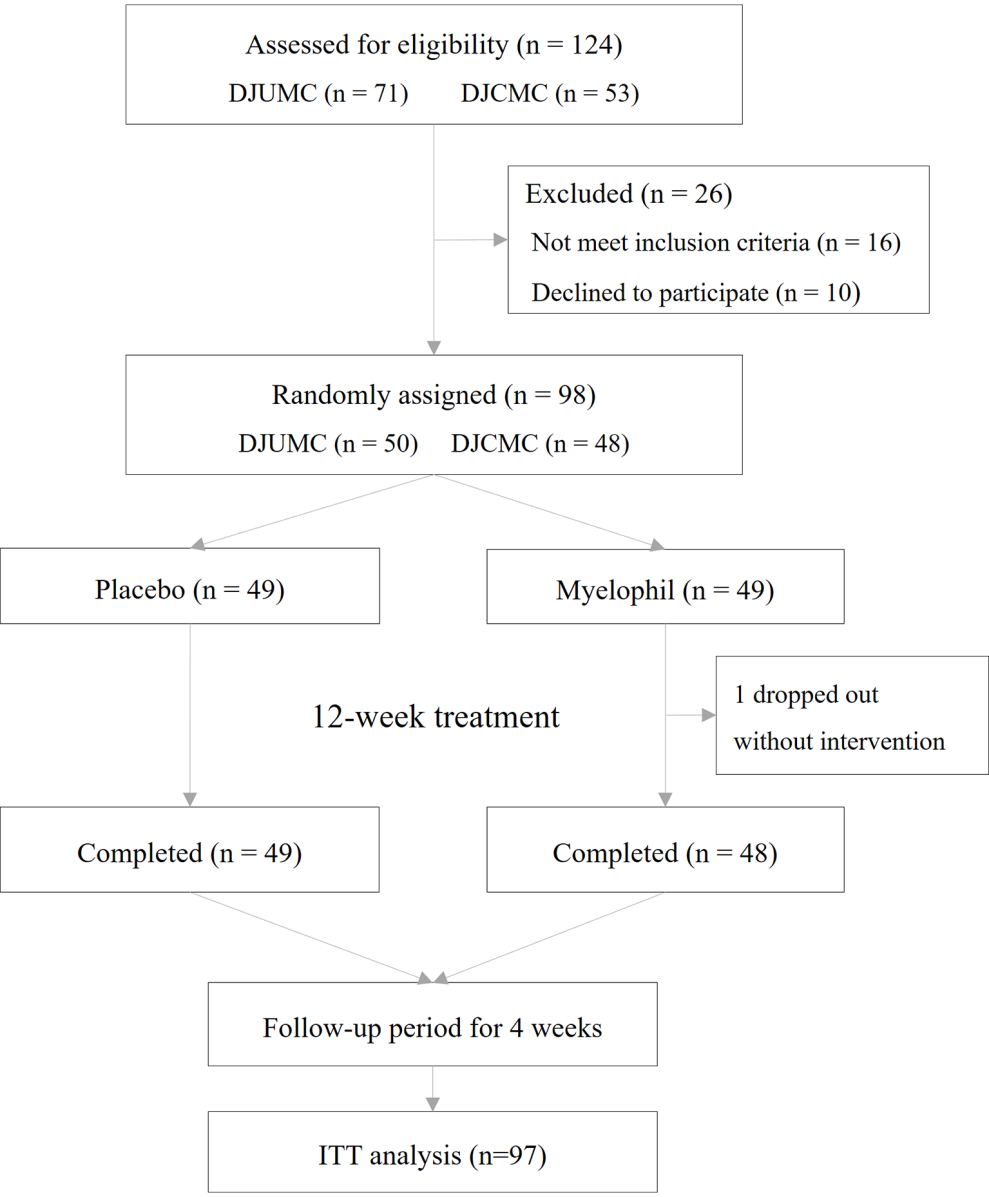
Randomized controlled trial flowchart. Illustration of study design and participant flow.

This study was a double-blind, placebo-controlled, randomized, two-center trial. Patients who received a diagnosis of CFS were recruited at a 1:1 ratio from the Daejeon Korean Medicine Hospital of Daejeon University and Daejeon St. Mary’s Hospital of the Catholic University of Korea. Eligible patients were randomly assigned at a 1:1 ratio to receive Myelophil at a dose of 2 g orally per day or matching placebo pills in 2 doses for 12 weeks. The target dose was determined based on preclinical studies and animal toxicity studies. In animal experiments using mice, Myelophil showed optimal effects at doses above 200 mg/kg/day (Kim et al., 2014; Lee et al., 2014). The human no-observed-adverse-effect level (NOAEL) value that was estimated by animal toxicity tests using rodents and non-rodents (beagle dog) was 694 mg/kg (Jung et al., 2009; Joung et al., 2019). All patients, investigators, and research assistants were blinded to the study-group assignments. The random allocation number was handled by independent statistical experts, and blocked random assignments were applied. Once a month, any unconsumed medications were returned to measure the medication adherence. The study flow chart is shown in **Figure 1**.

### Preparation and Standardization of Myelophil

**Figure 2:**
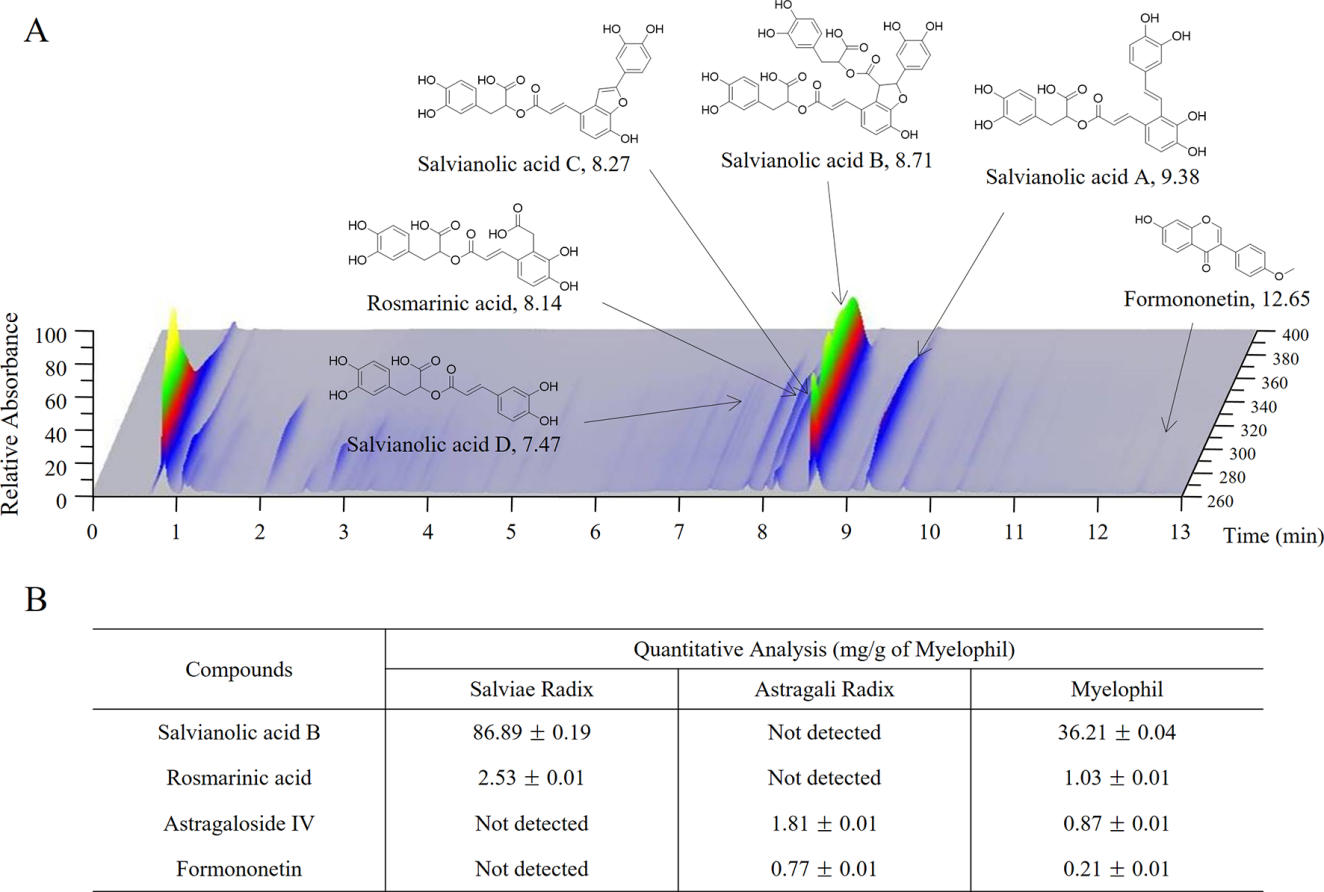
UHPLC and LC/MS chromatogram of Myelophil. **(A)** Myelophil and five standards were subjected to UHPLC analysis. **(B)** Myelophil and four major compounds were quantitative analyzed by LC/MS.

Myelophil was prepared in capsule form by Kyongbang Pharmacy according to Korean Good Manufacturing Practice guidelines (Korea Food and Drug Administration notification no. 2015-35). Myelophil is the 1:1 mixture of *Astragali Radix* and *Salviae Miltiorrhizae Radix* and was extracted using 30% ethanol for 20 h at 80°C. In preclinical studies, we found that 30% ethanol extract has more therapeutic effect than aqueous extract (data not shown). The final product that was obtained with a yield of 20.52% (w/w) was stored for future use. One capsule of Myelophil was made by extracting 1.389 g of *Astragali Radix* and 1.389 g of *Salviae Radix* with ethanol, and its weight was 600 mg. Molecular fingerprinting of Myelophil (**Figure 2A**) was conducted by ultra-high performance liquid chromatography tandem mass spectrometry (UHPLC–MS/MS, Thermo Scientific, San Jose, CA, USA) as described previously (Lee et al., 2014). For quantitative analysis, liquid chromatography–mass spectrometry (LC/MS, LTQ Orbitrap XL linear ion-trap MS system, Thermo Scientific Co., San Jose, CA, USA) (**Figure 2B**) was also conducted on the Myelophil and four reference compounds (stragaloside IV and formononetin for *Astragali Radix* and salvianolic acid B and rosmarinic acid for *Salviae miltiorrhizae Radix*, respectively) as previously described (Lee et al., 2015). The matching placebo was also prepared by the Kyongbang Pharmacy, and it contained a starch and lactose mixture of the same size, weight, and shape as Myelophil.

### Study Procedures, and End Points

We conducted screening tests on the participants, including an abdominal ultrasonography, chest X-ray, thyroid function test, State-Trait Anxiety Inventory (STAI) test, and Beck Depression Inventory (BDI) test, to rule out other diseases. Participants were evaluated at baseline and every 4 weeks until week 12. The primary end point was a change in the numeric rating scale (NRS) of the Chalder fatigue scale after a 12-week treatment. The Chalder fatigue scale is an 11-item symptom questionnaire that was created by Trudie Chalder and is used to measure the severity of both physical (questions 1–7) and mental (questions 8–11) tiredness in fatigue illnesses (Chalder et al., 1993; Cella and Chalder, 2010). In this study, the questionnaire was translated into Korean and then slightly modified by the NRS method to evaluate the fatigue severity in detail. All participants scored each item on a 10-point scale (0 = not at all to 9 = unbearably severe condition) as a self-rating numeric scale (total score range 0–99). The changes in physical and mental fatigue were also analyzed by dividing the items of the Chalder fatigue scale. The severity of physical fatigue was scored as physical NRS (sum of questions 1–7), and the severity of mental fatigue was scored as mental NRS (sum of questions 8–11). The modified questionnaire evaluation was appropriately applied in previous studies (Cho et al., 2009; Lee and Son, 2012).

The secondary outcomes included changes in fatigue severity from baseline, as measured by the visual analogue scale (VAS), fatigue severity scale (FSS), health-related quality of life, blood biomarkers of oxidative stress, and blood cytokines. The VAS was assessed by asking the patient to specify their level of overall discomfort from CFS by indicating a position along a continuous 10-cm line between two end points (Khanna et al., 2008). The FSS is a seven-point Likert scale that consists of nine items to easily measure physical fatigue (Krupp et al., 1989; Kim et al., 2016). The health-related quality of life was measured using a 36-item Short-Form Health Survey (SF-36), in which higher scores indicate a better quality of life (Myers and Wilks, 1999). Recent studies have shown oxidative stress and cytokines to be involved in CFS (Nakamura et al., 2010; Fukuda et al., 2016). This study analyzed levels of oxidative (serum reactive oxygen species and malondialdehyde) and antioxidative [total antioxidant capacity, catalase, superoxide dismutase, total glutathione (GSH), glutathione peroxidase (GSH-Px), and glutathione reductase (GSH-Rx)] indicators and cytokines [tumor necrosis factor-alpha (TNF-α) and interferon-gamma (IFN-γ)] in the blood before and after treatment. The safety of Myelophil was assessed through hematological analyses that included a liver function test, renal function test, complete blood count, and urinalysis. Adverse events were also observed during the trial. The timing of these evaluations is described in **Supplementary Table 3**.

### Sample Size Estimation

This study aimed to assess the efficacy of Myelophil in patients with CFS. The formula for estimating the sample size for each arm is as follows:

$$\text{n}=\frac{\left\{{\left(z_{\alpha /2}+z_{\beta }\right)}^{2}\sigma^{2}(\lambda +1)/\lambda \right\}}{{\left(\mu_{c}-\mu_{\tau }\right)}^{2}}$$

Based on the previous pilot study, we assumed that the mean value of fatigue indicated by the NRS would decrease by 6 in the placebo group and by 12 in the Myelophil group (µ*_c_* − µ*_t_* = 6). The standard deviation (s. d. = σ) of the change in NRS was assumed to be 8.8. In this study, the ratio of the treatment group to the placebo group was 2:1 (λ = 1). With a statistical power of 85% and a significance level (*a*) at 5%, 39 patients were required for each study arm. Assuming that 20% of patients would drop out or be lost to follow-up, this study would recruit 49 participants to each group, totaling 98 participants.

### Statistical Analysis

The analysis was conducted on the intention-to-treat (ITT) population, which included all patients who had a complete baseline assessment and received at least one dose of the study drug in the treatment phase but excluded patients who did not receive any study medications for various reasons. A per-protocol analysis was performed as a sensitivity analysis and included those patients who completed the trial and had at least 75% adherence. If necessary, post hoc subgroup analyses were performed according to gender, hospital, age, or fatigue severity. A safety analysis was performed on all patients who received at least one dose of the trial medication. The continuous variables, including the primary end points, were compared using an independent t-test. Categorical variables such as demographic characteristics were compared using Pearson’s chi-square test or Fisher’s exact test. The changes of variables over time within each group were compared using a paired t-test. Statistical significance was considered to be indicated by a p-value less than 0.05.

## Results

### Characteristics of the Participants

This phase 2 trial was conducted from December 2016 to November 2017 in two hospitals where 124 preliminary candidates were recruited. Of these, 98 consecutive participants who fulfilled the inclusion criteria were enrolled in this study. One participant dropped out due to personal reasons before starting the first drug administration and was excluded from the ITT analysis. In total, 97 patients (37 male and 60 female) were included in the ITT analysis, of which 48 were assigned to receive Myelophil and 49 to receive a placebo. The baseline demographics and clinical characteristics were similar between the two trial groups. The age (means ± SD) of participants was 39.7 ± 10.0 years. The BDI and STAI scores were 4.0 ± 4.7 and 48.3 ± 5.8, respectively (**Table 1**). All patients included in the ITT analysis completed a 12-week clinical trial with greater than 75% adherence. Thus, the per-protocol analysis was not performed because it would be identical to the ITT analysis.

**Table 1:** ITT Population baseline demographic and clinical characteristic.

Variables	Placebo (n = 49)	Myelophil (n = 48)	Total (%)
Median age (range)	39.5 ± 11.1 (21–64)	39.8 ± 8.7 (23–58)	39.7 ± 10.0 (21–64)
Gender			
Male	19	18	37 (38.1%)
Female	30	30	60 (61.9%)
Total	49	48	97 (100)
BMI (kg/m^2^)	22.5 ± 2.5	22.7 ± 2.8	22.6 ± 2.6
Hospital			
DJOMC (M:F)	25 (10/15)	24 (11/13)	49 (21/28)
DJCMC (M:F)	24 (9/15)	24 (7/17)	48 (16/32)
Total (M:F)	49 (19/30)	49 (19/30)	97 (37/60)
Chalder NRS			
Total	62.4 ± 13.5	61.6 ± 17.5	62.0 ± 15.5
Physical	41.5 ± 7.8	42.3 ± 10.4	42.3 ± 9.1
Mental	20.7 ± 6.8	20.0 ± 8.0	19.9 ± 7.4
VAS	7.2 ± 1.9	6.9 ± 1.6	7.1 ± 1.7
FSS	45.7 ± 7.5	45.4 ± 11.9	45.5 ± 9.9
SF-36	92.5 ± 15.0	90.4 ± 17.0	89.3 ± 16.0
BDI	2.1 ± 4.7	3.9 ± 4.6	4.0 ± 4.7
STAI	45.7 ± 5.0	49.1 ± 6.4	48.3 ± 5.8

Values are mean ± standard deviation or number (%). NRS, Numeric Rating Scale; VAS, Visual Analogue Scale; FSS, Fatigue Severity Scale; SF-36, 36-item Short-Form Health Survey; BDI, Beck Depression Inventory test; STAI, State-Trait Anxiety Inventory test.

### Changes in Numeric Rating Scale

**Figure 3:**
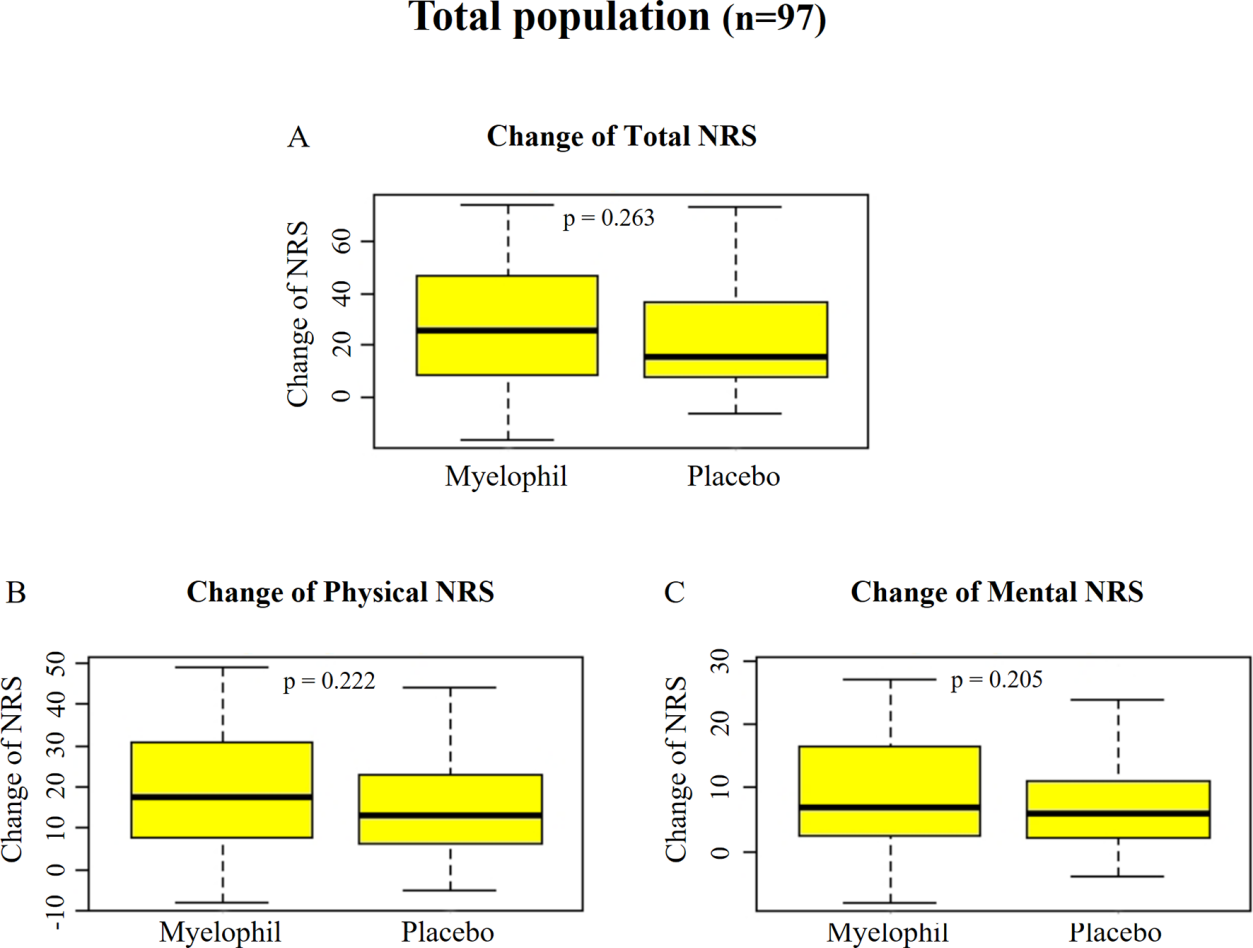
Changes of NRS before and after the treatment. **(A)** Total NRS. **(B)** Physical NRS. **(C)** Mental NRS. Independent t-test were used for group comparison. NRS, Numeric Rating Scale.

The initial NRS scores (means ± SD) were uniformly distributed at 61.8 ± 17.4 and 62.4 ± 13.5 in the Myelophil and placebo groups, respectively. After the 12-week treatment, the NRS scores of both groups were reduced to 34.8 ± 16.4 in the Myelophil group and 40.53 ± 19.0 in the placebo group (the score changes in the Myelophil and placebo groups: 26.6 ± 22.5 *vs*. 21.9 ± 18.3, respectively, P = 0.263, **Figure 3A**). The physical and mental NRS scores decreased in both groups (physical 17.9 ± 14.0 *vs*. 14.7 ± 11.8 and mental 9.1 ± 9.2 *vs*. 6.9 ± 7.1) but were not statistically significant (**Figures 3B, C**).

### Changes in Numeric Rating Scale by Subgroup (Numeric Rating Scale ≥63)

**Figure 4:**
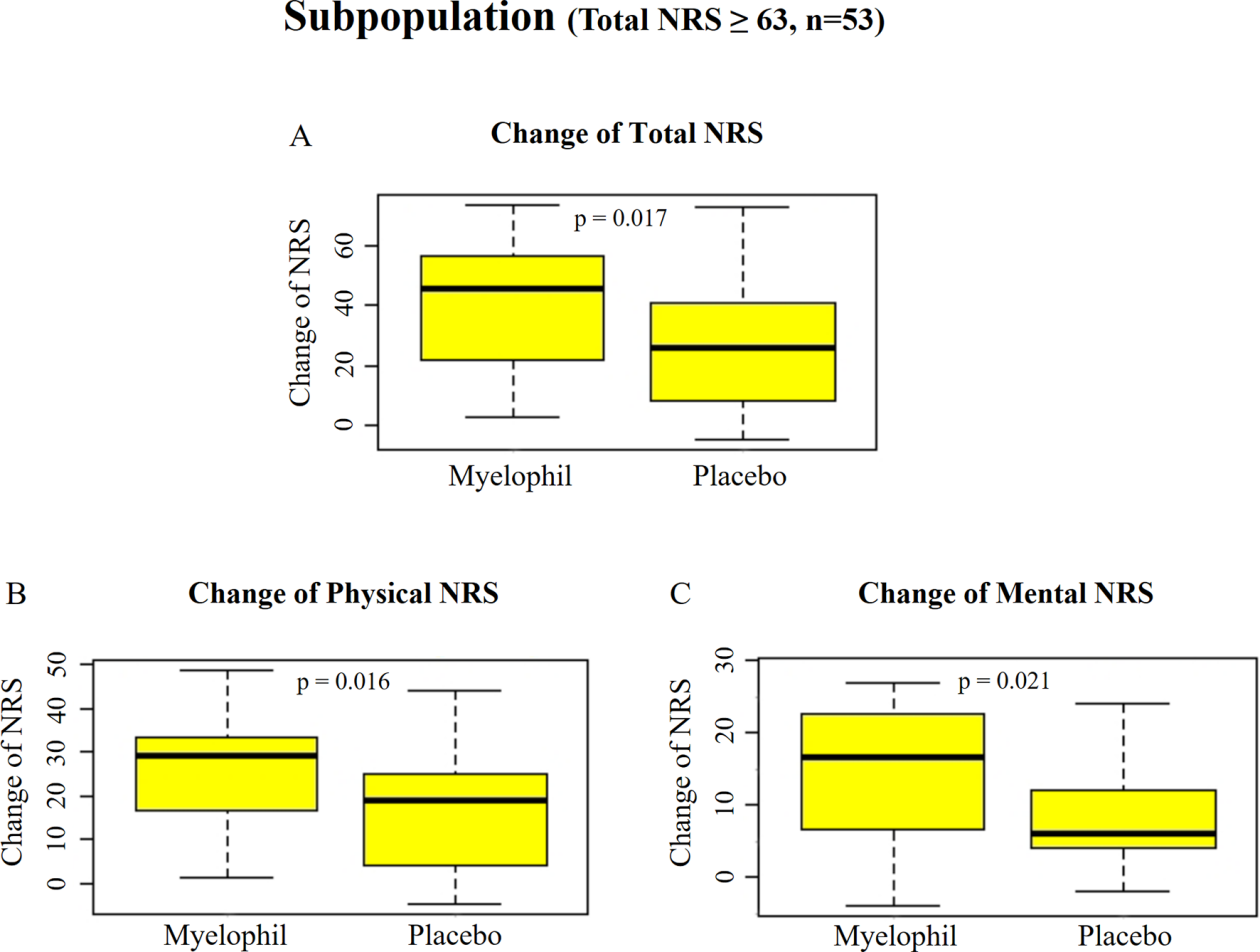
Changes of NRS before and after the treatment in subpopulation with severe fatigue (total NRS ≥ 63). **(A)** Total NRS. **(B)** Physical NRS. **(C)** Mental NRS. Independent t-test were used for group comparison. NRS, Numeric Rating Scale.

Post hoc subgroup analyses were performed to identify a subgroup in which Myelophil might show better efficacy. We focused on a subgroup with severe symptom (NRS ≥ 63, which was the median value of all subjects) that involved 53 participants (24 in Myelophil and 29 in placebo; M: 23, F: 30). The initial NRS of this subgroup was slightly higher in the Myelophil group (75.3 ± 8.1) than in the placebo group (71.0 ± 5.8). After the 12-week treatment, the NRS scores were reduced to 35.8 ± 17.9 in the Myelophil group and 45.3 ± 19.7 in the placebo group, at which point the changed value was significant (39.5 ± 21.0 *vs*. 25.7 ± 19.6, p = 0.017, **Figure 4A**). Both the physical and mental NRS scores were significantly improved (physical 25.3 ± 12.6 *vs*. 16.5 ± 12.8, p = 0.016 **Figure 4B** and mental 14.3 ± 9.1 *vs*. 8.9 ± 7.4, p = 0.021 **Figure 4C**). In other subgroup analyses (gender, hospital, and age), no significant difference was observed between Myelophil and placebo groups (data not shown).

### Changes in Visual Analogue Scale

**Figure 5:**
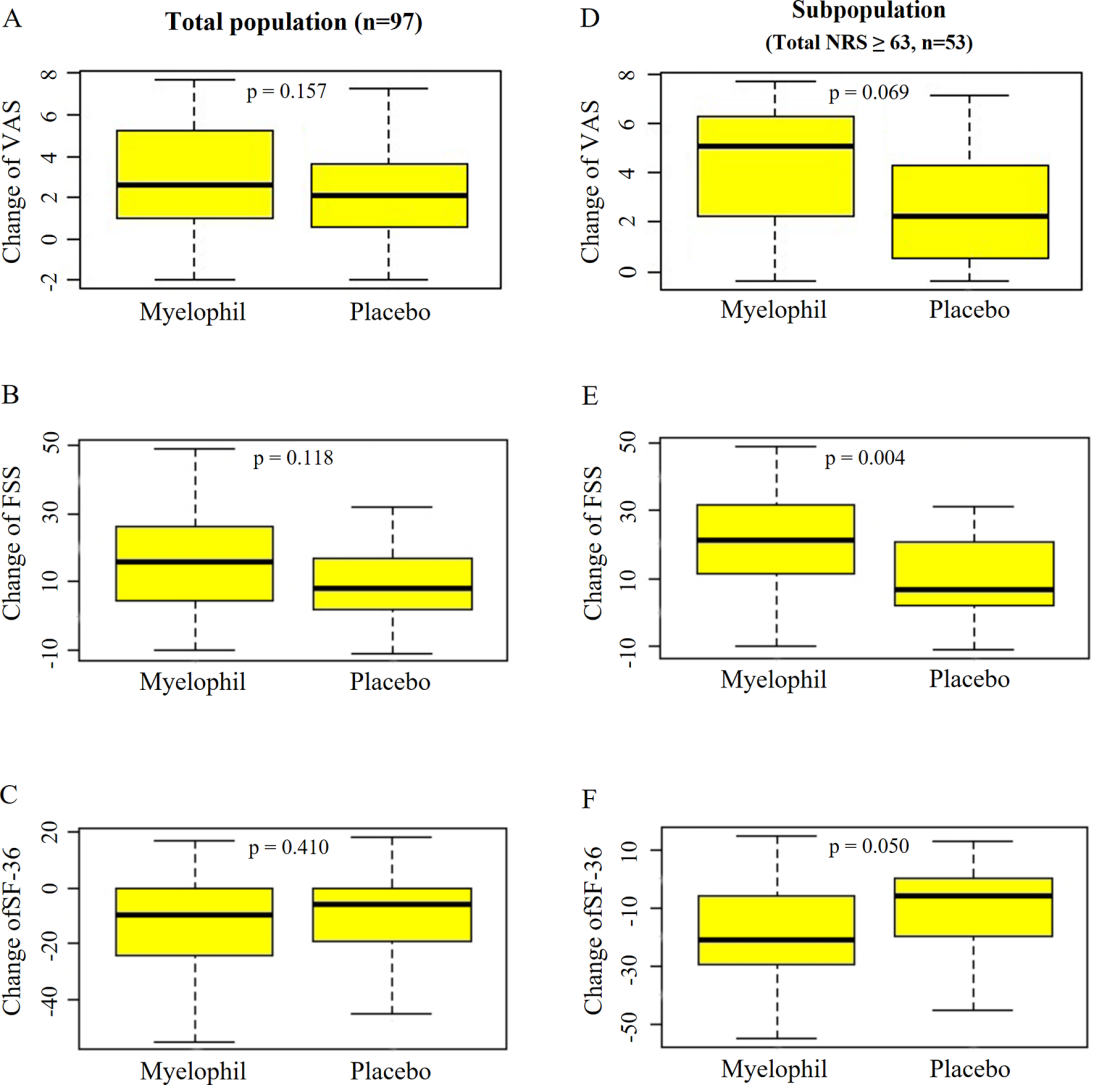
Changes of VAS, FSS, and SF-36 before and after the treatment. **(A)** VAS, **(B)** FSS, and **(C)** SF-36 in total population. **(D)** VAS, **(E)** FSS, and **(F)** SF-36 in subpopulation with severe fatigue (total NRS ≥ 63). Independent t-test were used for group comparison. VAS, Visual Analogue Scale; FSS, Fatigue Severity Scale; SF-36, 36-item Short-Form Health Survey.

The baseline VAS was 6.9 ± 1.6 in the Myelophil and 7.3 ± 1.9 in the placebo group. The VAS score was reduced in both groups (3.0 ± 2.4 *vs*. 2.5 ± 2.3, p = 0.157, **Figure 5A**) after the 12-week treatment. In the subgroup with severe CFS, the baseline VAS measurements were 7.8 ± 0.9 and 7.4 ± 0.8 in the Myelophil and placebo groups, respectively. The VAS was decreased in the Myelophil group compared with that in the placebo group, with a distinct trend toward significance (4.2 ± 2.5 *vs*. 2.8 ± 2.5, p = 0.069. **Figure 5D**).

### Changes in FSS

At baseline, the FSS scores were 45.4 ± 11.8 and 45.7 ± 7.5 in the Myelophil and placebo groups, respectively. After the 12-week treatment, the FSS score decreased in both group (15.3 ± 14.3 *vs*. 11.1 ± 11.6, P = 0.118, **Figure 5B**). In the analysis of a subgroup with severe CFS, the FSS was significantly reduced in the Myelophil group compared with that in the placebo group (21.7 ± 14.2 *vs*. 10.7 ± 11.9, p = 0.004, **Figure 5E**) and compared with their initial scores (52.8 ± 7.4 and 48.6 ± 5.5).

### Changes in 36-item Short-Form Health Survey

The initial SF-36 scores were calculated as 91.3 ± 16.6 and 88.2 ± 15.0 in the Myelophil and placebo groups, respectively. After the 12-week treatment, the SF-36 score was reduced in both groups (12.9 ± 18.0 *vs*. 10.0 ± 16.5, p = 0.410, **Figure 5C**). In the severe CFS subgroup, the baseline SF-36 scores were 82.3 ± 15.7 and 86.0 ± 15.2 in the Myelophil and placebo groups, respectively, while they significantly increased after 12 weeks by 19.9 ± 18.5 *vs*. 10.4 ± 15.6, respectively (p = 0.050, **Figure 5F**).

### Parameters of Oxidative Stress and Cytokines

The serum samples from 97 participants were analyzed before and after treatment. There was no significant difference in the levels of oxidative indicators (serum reactive oxygen species and malondialdehyde), antioxidative indicators (total antioxidant capacity, catalase, superoxide dismutase, total GSH, GSH-Px, and GSH-Rd), and cytokines (TNF-α and IFN-γ) between the two groups. A post hoc subgroup analysis also did not show any significant differences. Details are shown in **Supplementary Table 4**.

### Safety

We analyzed the adverse events and hematological tests in 97 participants. Adverse events were reported in 14.6% of patients receiving Myelophil (7/48) and in 18.4% of those receiving the placebo (9/49). No significant difference was seen between the two groups. There were no serious adverse events during the trial in either the Myelophil or placebo groups. A 43-year-old female in the Myelophil group presented with anemia (hemoglobin 8.7 g/dl). Her hemoglobin level recovered without any treatment during a monitoring period of 8 weeks, and this was thought to be independent of Myelophil treatment. All of the adverse events disappeared after a short period. Detailed descriptions of the adverse events in each group are listed in **Table 2**.

**Table 2:** Adverse effects in participants.

Group	Participant (Sex/age)	Complains	Treatment period (weeks)	Outcome
Placebo	F/24	Vaginitis	4	Lost
	M/35	Finger pain	8	Lost
	F/24	Dyspepsia, fatigue	4	Lost
	M/63	Sore throat	8	Lost
	M/48	Sore throat	4	Lost
	F/40	Cervical abrasion	4	Lost
	F/50	Shingles, periodontitis	4	Lost
	M/28	Lymphadenopathy	8	Lost
	F/23	Elevated liver enzymes (AST: 139 IU/l, ALT: 246 IU/l, GGT: 75 IU/l)	12	Lost
Myelophil	M/66	Diarrhea, knee pain	12	Lost
	M/50	Common cold	4	Lost
	F/39	Migraine, neck pain	5	Lost
	M/48	Diarrhea	4	Lost
	F/28	Pulpitis, diarrhea	4 12	Lost
	M/38	Cough	8 weeks after end of the test	Lost
	F/43	Anemia (Hg 8.7 g/dl)	12	Lost

## Discussion

Although notable progress has been made in governmental-derived investments in CFS, especially in the US and UK, there is still strong need for further studies of CFS (Bested and Marshall, 2015). A meta-analysis reported that the global prevalence of CFS was 0.76–3.28% (dependent upon the assessment method) (Johnston et al., 2013). In the previous epidemiological studies that were conducted in Korea, the prevalence of CFS, as determined by the CDC criteria, was 0.6–1.2% (Kim et al., 2000, Kim et al., 2005). To date, the pathological mechanism is not understood; therefore, there are no standardized therapeutics for CFS globally (Griffith and Zarrouf, 2008).

By contrast, patients with medically unexplained fatigue tend to use alternative therapies such as herbal medicines to treat their debilitating conditions (Jones et al., 2007). Due to the presence of multiple compounds and multiple targets, there is increasing evidence to show that herbal remedies are suitable strategies for chronic fatigue disorders (Efferth and Koch, 2011). Since 2002, Myelophil has been used as an herb-derived remedy to treat fatigue-related disorders in Korea. Myelophil has been shown to have effective outcomes in patients with chronic fatigue-associated disorders such as ICF or CFS (Cho et al., 2009). Preclinical studies showed that Myelophil has pharmacological properties that allow it to prevent the neurons in the brain from stress-derived damage and to relieve fatigue and cognitive impairment via regulation of HPA axis, inhibition of neuroinflammation, and modulation of cholinergic activity (Kim et al., 2013, Kim et al., 2014; Lee et al., 2014).

To our knowledge, this study is the first randomized, double-blind, placebo-controlled trial for medication to treat CFS in Korea. This phase 2 trial was conducted from December 2016 to November 2017 in two hospitals in Daejeon City, South Korea. The present trial was aimed at investigating the clinical efficacy and safety of Myelophil for CFS and to provide the essential clinical data for the phase 3 trial. In this study, a total of 98 participants (38 males and 60 females) who suffered from chronic fatigue for over 6 months and presented with CFS-related symptoms were enrolled. Among them, one participant dropped out before starting the first drug administration and was excluded from the ITT analysis. Ninety-seven participants completed the trial. Our trial showed a high prevalence of working age (30–55 years old, 77% of total) and in women (1.6-fold more than males) as previous studies (Nacul et al., 2011; Bakken et al., 2014; Collin et al., 2017).

In this study, we used the Chalder FSS as a primary end point, which is an 11-item, 4-point scale questionnaire (total score range: 0–33) (Jackson, 2015). This fatigue assessment tool has been used in many studies of fatigue-associated clinical trials (Crawley et al., 2013; Ng and Yiu, 2013). We herein used a slightly modified 10-point scale NRS version (total score range: 0–99) for a more accurate evaluation. Our previous study found that this modification was well adapted to differentiate between healthy controls and subjects with ICF (Cho et al., 2009; Lee and Son, 2012). Myelophil administration for 12 weeks seemed to further improve the fatigue symptoms in CFS patients compared with the placebo; however, this difference did not reach statistical significance. Similar patterns were shown in the physical and mental NRS, VAS, FSS, and SF-36 assessments.

We have speculated on the potential reasons why Myelophil did not show a significant therapeutic effect. First, a high placebo effect can mask the efficacy of Myelophil. Our study showed a high placebo response rate of over 30%, whereas a previous meta-analysis reported that the placebo response rate of CFS patients was, on average, 20% (Cho et al., 2005). Many Korean people believe that traditional herbal medicines are beneficial for fatigue, which attributed to the high placebo effect in this study. Second, patient bias may have occurred. We enrolled the participants by the CDC criteria based on their answers to the yes/no questions. Since these criteria could not measure the severity of patients’ illnesses, the patient population may have included participants with mild levels of symptoms. Generally, ICF is a less serious condition than CFS (Janse et al., 2016). In our previous study, the average NRS score of patients with ICF was 53.0 ± 15.1 (Lee and Son, 2012), as compared with 61.9 ± 15.5 in the present study. Approximately 25% of the participants in this present study reported an NRS score that was lower than the average NRS score of patients with ICF. With this background, we performed a post hoc analysis in a subpopulation consisting of 53 participants whose fatigue severity was equal to or greater than the median value of 63. The analysis of this subpopulation has two advantages. First, the high placebo effect can be suppressed. In Randomized controlled trial studies including CNS-related disorders, the drug–placebo difference tends to be greater in patients with severe symptoms (Khin et al., 2011; Schmidt et al., 2013; Weimer et al., 2015). Second, the participants who have fatigue that is too mild for them to receive treatment can be excluded, which increases the consistency of sample. A previous phase 3 trial of rintatolimod (an immune modulator) for CFS was performed in patients with severe CFS (Strayer et al., 2012).

Interestingly, the subpopulation analysis showed a significantly beneficial effect of Myelophil on CFS symptoms compared with the placebo (1.5-fold higher than placebo). This statistical significance was also obtained when the fatigue was analyzed for both the physical and mental fatigue aspects. Positive results were also shown in both the FSS and SF-36 measurements. In the analysis for VAS, there was a pattern of improvement with a considerable trend toward significance. These results were consistent in all fatigue-related assessments and supported the potential efficacy of Myelophil for the management of CFS.

In the pathophysiological aspects, oxidative stress and inflammatory cytokines are well recognized as potential contributors to the fatigue-associated disorders (Maes et al., 2011; Montoya et al., 2017). To support the patient-derived subjective assessments, eight oxidative stress parameters and two immunological cytokines (TNF-α and IFN-γ) were examined in this trial. Unfortunately, these parameters did not show any significant change in either the total population or the subpopulation. This result could be interpreted as follows: the relevant indices in the blood test are not appropriate as assessment variables for CFS. In previous studies, immunological indicators of CFS patients have often been found to be inconsistent. For example, one study reported an increased level of interleukin-8 in patients with CFS (White et al., 2010), but another study reported its decrease (Fletcher et al., 2009). Since cytokines or immunological factors are characterized by cyclical secretion, it is difficult to reflect the severity of CFS (Peterson et al., 2015). However, controversies surrounding the relationship between immune factors and CFS still remain. Many studies have reported that various inflammatory cytokines and oxidative stress can exhibit a linear relationship with disease severity (Maes et al., 2012; Shungu et al., 2012). In fact, the present study showed a positive trend in changes of TNF-α and IFN-γ with disease severity, but this was not statistically significant. Recently, a meta-analysis showed that serum level of TGF-β was significantly higher in CFS patients than that in healthy control subjects (Montoya et al., 2017); unfortunately, this was not measured in our study. TGF-β is undoubtedly a secondary parameter that should be included in our next clinical trial.

With respect to safety, there appeared to be no notable safety issues during the trial, as measured by serum chemistry, hematology, or urinalysis. Three out of 48 patients in the Myelophil group experienced mild diarrhea. All of these patients reported intermittent diarrhea before their participation in the trial, and they recovered from these symptoms within a short period without any treatment, so we concluded that this side effect was not related to Myelophil administration. Based on previous and the current clinical studies (Cho et al., 2009) and preclinical tests of repeated toxicity and genotoxicity (Jung et al., 2009; Lee et al., 2018), Myelophil is assumed to be safe. However, the risk of clinical long-term use should be further investigated.

The present study has the following limitations: 1) the initially specified analysis of the entire group did not show the significant therapeutic benefits. The post hoc subgroup analysis gives a general limitation of the clinical study (Schühlen, 2014). 2) At present, the Multidimensional Fatigue Inventory (MFI) is well applied in many clinical studies worldwide (Arnold et al., 2015; Maness et al., 2019). The MFI has five categories to evaluate multidimensional aspects such as reduced activity, cognitive impairment, and declining motivation, and the Korean version of MFI has been verified for its reliability and validity (Song et al., 2018). Accordingly, we need to consider the MFI as the primary end point and participants with moderate and severe symptoms of CFS in the next phase 3 trial with Myelophil.

## Conclusion

In conclusion, the results of this study reveal that Myelophil can be well tolerated for at least 3 months of oral administration and is a potential therapeutic candidate in CFS patients, especially those who have a severe symptom. This study indicates the rationale for the progression to a phase 3 trial with a larger sample size and further defined participant criteria.

## Data Availability

The raw data supporting the conclusions of this manuscript will be made available by the authors, without undue reservation, to any qualified researcher.

## Ethics Statement

This study was carried out in accordance with the rules, regulations, and guidelines of Ministry of Food and Drug Safety of Republic of Korea with written informed consent from all subjects. All subjects gave written informed consent in accordance with the Declaration of Helsinki. The protocol was approved by the institutional review board of Daejeon Korean Medicine Hospital of Daejeon University and Daejeon St. Mary’s Hospital of the Catholic University of Korea.

## Author Contributions

C-GS and D-SL conceived and designed the study. J-YJ and J-SL performed the study. Y-CA analyzed the data. J-YJ and C-GS wrote the manuscript. J-HC revised the manuscript. All of the authors approved the final version of the manuscript and agreed to be accountable for all aspects of this work.

## Funding

This study was supported by a grant from the Traditional Korean Medicine R&D Project, Ministry of Health & Welfare, South Korea (HI15C0112) and by the Ministry of Education, Science and Technology, South Korea (NRF-2018R1A6A1A03025221).

## Conflict of Interest Statement

The authors declare that the research was conducted in the absence of any commercial or financial relationships that could be construed as a potential conflict of interest.
