# Supplementary material for: The Efficacy and Safety of Myelophil, an Ethanol Extract Mixture of Astragali Radix and Salviae Radix, for Chronic Fatigue Syndrome: A Randomized Clinical Trial
Source: Front Pharmacol. 2019 Sep 10;10:991. doi: 10.3389/fphar.2019.00991 (PMC6746924; doi:10.3389/fphar.2019.00991)
Supplement: Supplementary file 1 [file Table_1.docx]

| **Supplementary Table 1. Inclusion criteria** |
| --- |
| The inclusion criteria are as follows |
| 1) Participants who are 18 - 65 years old |
| 2) Participants who meet the definition of CFS according to CDC Criteria.  Referred with a severe chronic fatigue for more than 6 months that is not alleviated by rest, and is not due to other medical conditions. Additionally, at least four of the following symptoms must be present |
| 1. Postexertional malaise lasting more than 24 hours 2. Unrefreshing sleep 3. Difficulty with thinking and memory 4. Muscle pain 5. Multiple joint pain without joint swelling or redness 6. Headache of a new type, pattern, or severity 7. Cervical or axillary lymphadenopathy 8. Sore throat |
| 3) Participants who can complete the relevant questionnaires in this trial |
| 4) Participants who can give informed consent to participate in this trial |
| CFS: Chronic Fatigue Syndrome, CDC: US Centers for Disease Control and Prevention |

| **Supplementary Table 2. Exclusion criteria** |
| --- |
| The exclusion criteria are as follows |
| 1) Participants who take medication for chronic illness.  2) Participants who have diseases that induce chronic fatigue within the past 6 months.  For example, hypothyroidism, anemia, or psychiatric disorders, etc.  3) Participants whose hemoglobin is lower than 12g/dL in men, 11g/dL in women.  4) Participants whose AST or ATL is more than 2 times the upper limit of normal,  or creatinine is more than 1.2 times the upper limit of normal.  5) Participants with high-grade fatty liver on ultrasonography.  6) Participants with a history of cancer.  7) Participants who work at night.  8) Participants who drink more than twice a week.  9) Participants whose BMI is less than 16.5 or more than 30.  10) Participants who have undergone organ transplantation or take immunosuppressive medication.  11) Participants who have been treated for chronic fatigue during the last 2 months.  For example, medication, cognitive behavioral or exercise therapy.  12) Woman who are pregnant or lactating. Woman who have tried to get pregnant  during childbearing age.  13) Participants whose BDI is more than 25  14) Participants whose STAI is more than 60.  15) Participants who cannot understand and cannot follow this trial. |
| BMI: Body Mass Index, BDI: Beck Depression Inventory), STAI: State-Trait Anxiety Inventory |

| **Supplementary Table 3. Time table of evaluation of the clinical trial** | | | | | | |
| --- | --- | --- | --- | --- | --- | --- |
| Evaluation | Screening | Treatment period (weeks) | | | | 8-week  follow up |
|  |  | 0 | 4 | 8 | 12 |  |
| Visit | 1 | 2 | 3 | 4 | 5 | 6 |
| Consent form | ◎ |  |  |  |  |  |
| Eligibility evaluation | ◎ |  |  |  |  |  |
| Demographic survey | ◎ |  |  |  |  |  |
| Vital signs | ◎ | ◎ | ◎ | ◎ | ◎ | ◎ |
| Physical examination | ◎ |  |  |  |  |  |
| Chalder NRS score | ◎ |  | ◎ | ◎ | ◎ | ◎ |
| Faigue VAS score | ◎ |  | ◎ | ◎ | ◎ | ◎ |
| FSS score | ◎ |  | ◎ | ◎ | ◎ | ◎ |
| Biomarkers of oxidative stress (blood) | ◎ |  |  |  | ◎ |  |
| Cytokines (blood) | ◎ |  |  |  | ◎ |  |
| SF-36 | ◎ |  |  |  | ◎ |  |
| STAI | ◎ |  |  |  |  |  |
| BDI | ◎ |  |  |  |  |  |
| Abdominal ultrasono | ◎ |  |  |  |  |  |
| Chest X-ray, EKG | ◎ |  |  |  |  |  |
| Pregnancy test | ◎ |  |  |  | ◎ |  |
| CBC, LFT, Urinalysis | ◎ |  | ◎ |  | ◎ |  |
| Hepatitis screening | ◎ |  |  |  |  |  |
| Thyroid function test | ◎ |  |  |  |  |  |
| BMI | ◎ |  |  |  | ◎ |  |
| Randomization |  | ◎ |  |  |  |  |
| Prescription |  | ◎ | ◎ | ◎ |  |  |
| Co-medication investigation |  | ◎ | ◎ | ◎ | ◎ | ◎ |
| Adverse events |  | ◎ | ◎ | ◎ | ◎ | ◎ |
| Medication adherence |  |  | ◎ | ◎ | ◎ |  |
| NRS: Numeric Rating Scale, VAS: Visual Analogue Scale, FSS: Fatigue Severity Scale, SF-36: Short-Form Health Survey, STAI: State-Trait Anxiety Inventory test, BDI: Beck Depression Inventory test, EKG: electrocardiogram, CBC: Complete Blood Count, LFT: Liver Function Test, BMI: Body Mass Index | | | | | | |

| **Supplementary Table 4. Change of the CFS related bio-marker in blood test** | | | | | |
| --- | --- | --- | --- | --- | --- |
| **Parameter** | **Week** | **Total participants (n=97)** | | **NRS ≥ 63 (n=53)** | |
|  |  | **Placebo** | **Myelophil** | **Placebo** | **Myelophil** |
| ROS | 0 | 33.0 ± 14.2 | 32.5 ± 12.7 | 33.7 ± 16.2 | 32.6 ± 14.8 |
|  | 12 | 28.4 ± 8.9 | 29.2 ± 9.1 | 26.5 ± 8.6 | 28.2 ± 9.9 |
| MDA | 0 | 0.37 ± 0.50 | 0.38 ± 0.36 | 0.41 ± 0.63 | 0.41 ± 0.45 |
|  | 12 | 0.29 ± 0.27 | 0.30 ± 0.40 | 0.25 ± 0.25 | 0.28 ± 0.38 |
| TAC | 0 | 0.10 ± 0.26 | 1.03 ± 0.26 | 0.99 ± 0.26 | 1.04 ± 0.29 |
|  | 12 | 0.90 ± 0.25 | 0.84 ± 0.20 | 0.93 ± 0.22 | 0.91 ± 0.18 |
| SOD | 0 | 169.5 ± 32.4 | 177.0 ± 42.4 | 161.9 ± 29.3 | 187.4 ± 57.0 |
|  | 12 | 176.1 ± 40.1 | 182.0 ± 48.8 | 169.0 ± 36.4 | 189.6 ± 64.8 |
| Catalase | 0 | 34.7 ± 19.4 | 35.2 ± 22.1 | 28.8 ± 18.1 | 34.3 ± 22.2 |
|  | 12 | 40.8 ± 27.7 | 35.9 ± 20.1 | 44.1 ± 28.5 | 38.0 ± 22.1 |
| GSH | 0 | 19.1 ± 8.6 | 18.8 ± 8.1 | 16.7 ± 7.0 | 16.5 ± 7.3 |
|  | 12 | 29.9 ± 8.6 | 28.7 ± 8.0 | 30.5 ± 13.0 | 28.7 ± 9.0 |
| GSH-Px | 0 | 73.9 ± 17.5 | 70.6 ± 19.0 | 70.7 ± 14.0 | 65.7 ± 18.3 |
|  | 12 | 74.9 ± 24.8 | 69.8 ± 17.6 | 70.9 ± 25.3 | 67.9 ± 17.7 |
| GSH-Rx | 0 | 10.0 ± 4.2 | 10.6 ± 3.5 | 9.2 ± 4.4 | 10.5 ± 3.4 |
|  | 12 | 10.3 ± 4.6 | 10.1 ± 4.8 | 10.4 ± 5.4 | 10.7 ± 5.2 |
| TNF-α | 0 | 1.07 ± 0.52 | 1.08 ± 0.58 | 1.26 ± 0.52 | 1.32 ± 0.61 |
|  | 12 | 0.93 ± 0.56 | 0.91 ± 0.44 | 1.11 ± 0.64 | 0.95 ± 0.35 |
| IFN-γ | 0 | 9.00 ± 3.58 | 8.68 ± 3.58 | 9.36 ± 3.34 | 8.28 ± 2.11 |
|  | 12 | 9.34 ± 3.66 | 8.22 ± 3.25 | 9.20 ± 3.35 | 8.73 ± 3.84 |
| Values are mean ± standard deviation. ROS: Reactive Oxygen Species, MDA: malondialdehyde, TAC: Total Antioxidant Capacity, SOD: Superoxide Dismutase, GSH: total glutathione, GSH-Px: glutathione peroxidase, GSH-Rx: glutathione reductase, TNF-α: Tumor Necrosis factor-alpha, IFN-γ: interferon-gamma | | | | | |
